# Occult Parathyroid Lesions on ^99m^Tc-Sestamibi Scintigraphy: Morphometric, Histopathological and Anatomical Determinants of Detection

**DOI:** 10.3390/biomedicines14071650

**Published:** 2026-07-22

**Authors:** Oriana-Eliana Pelineagră, Ioana Golu, Melania Balaș, Daniela Georgiana Amzăr, Iulia Plotuna, Oana Popa, Diana Aruncutean, Dan Cristian Roşu, Ion Icma, Agneta Maria Pusztai, Mărioara Cornianu, Mihaela Iacob, Nicu Olariu, Mihaela Vlad

**Affiliations:** 1 2nd Department of Internal Medicine—Discipline of Endocrinology, “Victor Babes” University of Medicine and Pharmacy, P-Ta Eftimie Murgu 2, 300041 Timisoara, Romania; oriana.pelineagra@umft.ro (O.-E.P.); balas.melania@umft.ro (M.B.); amzar.daniela@umft.ro (D.G.A.); iulia.plotuna@umft.ro (I.P.); oana.taban@umft.ro (O.P.); diana.aruncutean@umft.ro (D.A.); vlad.mihaela@umft.ro (M.V.); 2 Department of Endocrinology, County Emergency Hospital Timisoara, Blvd. Liviu Rebreanu 156, 300723 Timisoara, Romania; 3 Center for Molecular Research in Nephrology and Vascular Disease, “Victor Babes” University of Medicine and Pharmacy, P-Ta Eftimie Murgu 2, 300041 Timisoara, Romania; nicu.olariu@umft.ro; 4 2nd Department of Surgery—Discipline of Surgery I, “Victor Babes” University of Medicine and Pharmacy, P-Ta Eftimie Murgu 2, 300041 Timisoara, Romania; rosu.cristian@umft.ro; 5 1st Surgical Department, Pius Brînzeu Emergency County Hospital, 300723 Timisoara, Romania; nutu75@yahoo.com; 6 Department of Anatomy, “Victor Babes” University of Medicine and Pharmacy, P-Ta Eftimie Murgu 2, 300041 Timisoara, Romania; pusztai.agneta@umft.ro; 7 Nuclear Medicine Laboratory, Pius Brînzeu Emergency County Hospital, 300723 Timisoara, Romania; 8 Department of Microscopic Morphology—Anatomic Pathology, ANAPATMOL Research Center, “Victor Babes” University of Medicine and Pharmacy, Eftimie Murgu Square No. 2, 300041 Timisoara, Romania; cornianu.marioara@umft.ro; 9 Department of Pathology, “Pius Brinzeu” County Emergency Clinical Hospital, Bulevardul Liviu Rebreanu 156, 300723 Timisoara, Romania; iacobmiha@yahoo.com; 10 2nd Department of Internal Medicine—Discipline of Nephrology, “Victor Babes” University of Medicine and Pharmacy, P-Ta Eftimie Murgu 2, 300041 Timisoara, Romania; 11 Department of Nephrology, “Pius Brinzeu” County Emergency Clinical Hospital, Bulevardul Liviu Rebreanu 156, 300723 Timisoara, Romania

**Keywords:** parathyroid pathology, parathyroid scintigraphy, parathyroid burden, primary hyperparathyroidism, secondary hyperparathyroidism

## Abstract

**Background**: Accurate preoperative localization of hyperfunctioning parathyroid glands remains challenging, particularly in secondary hyperparathyroidism where multiglandular disease may compromise scintigraphic performance. **Methods**: Our study evaluated biochemical, morphometric, and histopathological predictors of ^99m^Tc-sestamibi scintigraphy detectability in both primary and secondary hyperparathyroidism. The study group included a total of 162 patients with primary and secondary hyperparathyroidism who underwent dual-phase ^99m^Tc-sestamibi scintigraphy followed by parathyroidectomy. Demographics, biochemical parameters, histopathological features, lesion volume and scintigraphic findings were assessed at patient and lesion level. **Results**: In primary hyperparathyroidism, adenomas were larger and more frequently detected than hyperplastic glands. Lesion volume and solid growth pattern were found as positive predictors of sestamibi uptake. In secondary hyperparathyroidism, nodular hyperplasia was associated with larger volume, higher cellularity, and more frequent localizing studies. Upper quadrant position and diffusely hyperplastic lesions were associated with higher lesion miss rates, while lesion volume increased the likelihood of detection. **Conclusions**: Our findings highlight that ^99m^Tc-sestamibi scintigraphy performance is strongly influenced by lesion volume, histopathological architecture and anatomical position, underscoring the needs for cautious interpretation of negative or incomplete scans, especially in secondary hyperparathyroidism.

## 1. Introduction

The parathyroid glands are small endocrine glands responsible for synthesis and secretion of parathyroid hormone (PTH), playing a vital role in calcium homeostasis. Embryologically, the superior parathyroid glands originate from the fourth pharyngeal pouch and subsequently migrate caudally to their final position adjacent to the superior pole of the thyroid gland, typically at the level of the cricoid cartilage [1]. In contrast, the inferior parathyroid glands have a different embryologic origin arising from the dorsal wing of the third pharyngeal pouch, whereas the thymus derives from its ventral component. During embryological descent, inferior glands migrate caudally and are typically located within 1 cm from the intersection of the inferior thyroid artery and the recurrent laryngeal nerve. This shared development pathway is fundamental in understanding the occurrence of ectopic parathyroids, which most frequently result from aberrant migration of the inferior glands along their trajectory with the thymus [2]. Ectopy involving the superior glands is considerably less frequent as their location is much more consistent. Nevertheless, ectopic locations have been reported in the carotid sheath, paralaryngeal space, anterior mediastinum, and tracheoesophageal groove as within the thyroidal parenchyma itself [3,4].

Based on biochemical parameters and patient history, hyperparathyroidism has been traditionally classified in one of three major forms: primary, secondary or tertiary hyperparathyroidism. Advances in the understanding of parathyroid pathophysiology have expanded this classification with other clinical variants, such as normocalcemic primary hyperparathyroidism, and more recently normohormonal primary hyperparathyroidism [5,6].

Primary hyperparathyroidism (PHPT) is characterized by autonomous hypersecretion of PTH, resulting in elevated serum calcium concentrations. Approximately 80–85% of cases of PHPT are attributed to a solitary parathyroid adenoma, whereas parathyroid hyperplasia affecting one or more parathyroid glands accounts for the remaining 10–15% of cases. Parathyroid carcinoma represents a rare cause for PHPT, being identified in only 0.5–5% of cases [2,3,7]. Histologically, adenomas are typically well-circumscribed lesions surrounded by a thin capsule and display a heterogeneous, often nodular, cut surface. Stromal fat is markedly reduced compared to normal parathyroid glands and some may retain a peripheral rim of normal or atrophic parathyroid tissue. Hyperplastic parathyroids tend to exhibit a more symmetric macroscopic appearance, featuring a homogenous cut-section while lacking the residual peripheral rim [8]. Parathyroid carcinomas are rare neoplasms affecting a single gland, but isolated cases of parathyroid carcinomas have been previously reported in patients with long-standing secondary hyperparathyroidism undergoing hemodialysis [9,10]. Atypical parathyroid adenomas, currently designated as atypical parathyroid neoplasia, demonstrate suspicious morphological features that raise concern for malignancy including an increased mitotic activity, atypical mitotic figures, presence of thick fibrous bands or calcifications [8]. The distinction between atypical adenomas and parathyroid carcinomas relies primarily on the absence of lymphovascular or perineural invasion and absence of distant metastasis [7].

Secondary hyperparathyroidism (SHPT) arises from excessive stimulation driven primarily by vitamin D deficiency, hyperphosphatemia and hypocalcemia. Several conditions including malnutrition, osteomalacia and chronic kidney disease (CKD) provide sustained stimuli for PTH secretion. Among these, end-stage CKD represents a particularly complex setting, characterized by excessive phosphate retention, impaired hydroxylation of 25-hydroxyvitamin D and subsequent hypocalcemia [11]. As glomerular filtration rate (GFR) declines, calcium phosphate homeostasis becomes disrupted and structural changes affecting the parathyroid gland become apparent. PTH levels begin to increase at a GFR of approximately 45 mL/min/1.73 $m^{2}$, whereas serum phosphate concentrations remain relatively stable upon reaching a GFR of approximately 20 mL/min/1.73 $m^{2}$ [12]. The reduction in functional nephron mass limits renal synthesis of 1,25-dihydroxyvitamin D. Concurrently, elevated serum phosphate alters PTH secretion via a posttranslational mechanism and further suppresses 1αhydroxylase activity, thereby further aggravating the PTH hypersecretion [13]. Histopathologically, the earliest alteration consists of diffuse parathyroid cell hyperplasia. In experimental murine models, this initial stage has been observed as early as 3–5 days after inducing uremia. Continued stimulation subsequently promotes the formation of nodular areas within the hypercellular parathyroid gland, a process recognized as nodular hyperplasia. Once nodular hyperplasia develops, resistance to medical therapy with vitamin D analogs, calcimimetics and phosphate binders becomes increasingly common, resulting in insuppressible PTH secretions and persistent hyperparathyroidism [12,14].

Tertiary hyperparathyroidism emerges when long-standing nodular hyperplasia acquires autonomous PTH secretion, which may persist despite reduction or attenuation of the initial stimulatory factors, as seen with renal transplantation. Although tertiary hyperparathyroidism is associated with elevated serum calcium concentrations, similarly to PHPT, tertiary hyperparathyroidism is readily distinguishable from PHPT based on patient history of prolonged SHPT, most often in the context of hemodialysis [6].

The management of primary hyperparathyroidism mainly involves surgical excision of the hyperfunctional gland after localization imaging. Greater therapeutic complexity arises in patients with parathyroid hyperplasia, in whom multiglandular involvement may result in persistent disease despite surgical intervention. In secondary hyperparathyroidism, current guidelines recommend maintaining a PTH value within two to nine times the upper limit of normal or <800 pg/mL [15]. When medical therapy becomes ineffective, surgical management is indicated, either as total parathyroidectomy followed by autotransplantation of 40–80 mg of parathyroid tissue, or as subtotal parathyroidectomy [3,15]. Preoperative localization studies are of critical importance especially in SHPT patients, as inaccurate identification or omission of hyperfunctioning glands can influence surgical outcome and results in persistent or recurrent hyperparathyroidism [16,17].

Accurate preoperative identification of abnormal parathyroid glands remains challenging, partly due to the reduced sensitivity for commonly used modalities, such as ultrasound and scintigraphy, and the restricted availability of more advanced investigations including fluoro-choline positron emission tomography (^18^F-FCH PET) or ^11^C-methionine PET [17,18]. Cervical ultrasound is accessible, non-radiating, and reproductible. However, it lacks specificity and is highly operator-dependent. Lymph nodes of the cervical region as well as certain thyroid nodules may closely mimic an abnormal parathyroid gland. Emerging evidence suggests that complementary investigations incorporated into conventional cervical ultrasound examination can benefit the differential diagnosis of parathyroid mimics. Parathyroid scintigraphy is another commonly available imaging technique and has been traditionally performed using ^99m^Tc-sestamibi, also known as methoxyisobutilisonitril. In recent years, substantial efforts have been focused on improving the diagnostic accuracy of sestamibi-based imaging for parathyroid lesions. The introduction of dual-tracer scintigraphy combining ^99m^Tc-sestamibi with either $I^{123}$ or ^99m^Tc-pertechnetate proved especially useful when concomitant thyroid pathology interferes with the results by delayed wash-out or intense uptake within thyroid nodules [19,20]. The addition of SPECT/CT further enhances spatial resolution and anatomical correlation. Recent studies have also supported the utility of 4D-computed tomography (4DCT) and 4D-magnetic resonance imaging (4DMRI) as sensitive imaging techniques, particularly for anatomical localization. However, their use may be limited by radiation exposure, motion-related artifacts and lower specificity compared to more advanced functional imaging techniques. Both ^18^F–FCH PET and ^11^C methionine PET have demonstrated good sensitivity and specificity, but their availability limits their routine use to only some centers. Dual-phase planar scintigraphy and SPECT/CT are routinely used and generally provide satisfactory performance in single-gland disease. In patients with multiglandular disease and negative or equivocal conventional imaging, ^18^F–FCH PET/CT may offer higher lesion-detection rates and improved spatial resolution, thereby providing additional value for preoperative surgical planning. False-positive findings may occur because of nonspecific tracer uptake in lymph nodes containing neoplastic tissue, although such cases appear to be uncommon [2,17,19]. The present study aims to bring additional insights into parathyroid gland pathology by identifying factors associated with non-localizing studies in both primary and secondary hyperparathyroidism.

## 2. Materials and Methods

We conducted a retrospective, observational study by reviewing the medical records of previously admitted patients in our hospital. Inclusion criteria comprised a diagnosis of primary or secondary hyperparathyroidism, complete biochemical data, available functional imaging, as well as histopathological results. Patients with incomplete data, missing imaging, those that did not undergo surgery or had an unsuccessful surgery with excision of a different structure other than a parathyroid gland were excluded. Data from 984 patients who underwent surgery for parathyroid lesions between 2016 and 2026 were retrospectively analyzed. Following the application of inclusion and exclusion criteria, a total of 162 patients were included in the study cohort.

Collected data included patients’ demographics and laboratory assessments. In patients diagnosed with secondary hyperparathyroidism undergoing hemodialysis, only the predialysis biochemical parameters were included in order to minimize the bias potentially related to dialysis timing. Demographics collected included age and gender, while laboratory assessments focused on total serum calcium, ionized serum calcium, serum phosphate, serum creatinine, iPTH and alkaline phosphatase. Serum creatinine (normal range: 0.6–1.0 mg/dL; 0.8–1.5 mg/dL), serum phosphate (normal range: 2.5–4.9 mg/dL; 2.5–4.5 mg/dL), total serum calcium (normal range: 8.5–10.1 mg/dL; 8.4–10.2 mg/dL) and alkaline phosphatase (normal range: 46–116 U/L; 38–126 U/L) were measured by spectrophotometry using Dimension_RXL_L4 (Siemens Healthcare Diagnostics, Newark, DE, USA) or Vitros 4600 (QuidelOrtho, San Diego, CA, USA). Ionized serum calcium (normal range: 4.2–5.2 mg/dL) was measured by spectrophotometry using Stream Lab (Stream Lab, San Francisco, CA, USA) or Attelica Solution CH1 (Siemens Healthineers, Malvern, PA, USA). Serum iPTH was measured using chemiluminescence on Immulite_2000XPI_L1 (Siemens Healthineers, Malvern, PA, USA) (normal range: 12–65 pg/mL) or Atellica Solution-IM (Siemens Healthineers, Malvern, PA, USA) (normal range: 18.5–88.0 pg/mL).

Histopathological examinations were performed by two experienced pathologists, and lesion-specific characteristics were collected. Each specimen was evaluated by only one pathologist and no independent double reading was performed; therefore, interobserver variability could not be assessed. During surgery all abnormal parathyroid glands were excised and sent for pathological evaluation. Lesion volume was calculated using a standardized ellipsoid formula (volume [mL] = π/6 × a × b × c). For patients with multiple lesions, cumulative parathyroid burden was defined as the sum of the volumes of all removed lesions in each individual patient. For each lesion, microscopic evaluation documented the presence or absence of intralesional calcifications, cystic degeneration, oxyphilic, chief cells and specific architectural patterns, including solid, acinar, trabecular, nested or pseudoadenomatous growth. Intralesional adipose content was assessed semi-quantitatively on a scale from 0 to 3, corresponding to absent, mild, moderate and abundant adipose tissue.

Functional imaging was performed in all included cases in the Nuclear Medicine Department of our hospital. All patients underwent dual-phase ^99m^Tc-sestamibi scintigraphy using methoxyisobutylisonitrile as radiotracer. Planar images were performed using a Nucline AP gamma camera, model MB9200 (Mediso Medical Imaging Systems Ltd., Szeged, Hungary), equipped with a NaI (Tl) scintillation crystal measuring 413 mm in diameter and 9.5 mm in thickness. The administered dose of ^99m^Tc-sestamibi ranged from 15 to 20 mCi and images were acquired at 20, 90 and 120 min after radiotracer administration, from an anterior projection including the neck and mediastinum. Low-energy high-resolution (LEHR) collimators were used with a 140 keV energy window and a 128 × 128 acquisition matrix. All scintigraphic examinations were interpreted by a single experienced nuclear medicine physician. Scintigraphic reports documented uptake intensity relative to the thyroid parenchyma in early (20 min) and delayed phases (90 and 120 min), as well as anatomical position in case of abnormal uptake. In our study, on an individual patient level, scintigraphy was considered positive only when all surgically removed hyperfunctioning glands were correctly identified. On a lesion level, a result was classified as positive when the excised gland corresponded anatomically to the position described on scintigraphy, even if additional glands from the same patient were missed. The nuclear medicine physician and the pathologists were blinded to the patients’ clinical history, biochemical parameters, and intraoperative findings.

Statistical analysis was performed using MedCalc^®^ Statistical Software version 23.5.9 (MedCalc Software Ltd., Ostend, Belgium), as well as JASP (Version 0.97.1; JASP Team (2026)) for graphics. Subgroup analysis included unpaired Student *t*-test, Mann–Whitney U test or Kruskal–Wallis test for numerical variables, while Χ-Square and Fisher exact test for categorical variables. The results were reported as mean ± standard deviation for Gaussian distributed variables or as median (IQR) for variables with a non-parametric distribution. Correlation tests were conducted using Spearman’s rank correlation. Logistic regression results were reported as overall model significance as well as individual variable odds ratio with 95% confidence intervals and statistical significance within multivariate analysis. Differences between groups were considered statistically significant if *p* < 0.05.

## 3. Results

### 3.1. General Study Cohort

A total of 162 patients diagnosed with primary or secondary hyperparathyroidism, admitted to our hospital between 2016 and 2026, met the inclusion criteria and were included in this study. The selected cohort was stratified into two different groups according to biochemical profile and clinical history. The PHPT group comprised 88 patients with elevated or normal serum calcium concentrations in association with increased PTH despite vitamin D supplementation, whereas the SHPT included 74 patients with end-stage chronic kidney disease undergoing hemodialysis and referred for surgical management of secondary hyperparathyroidism. Subgroup analyses were subsequently performed, and central tendency indicators and dispersion were reported for age, gender, total and ionized serum calcium, creatinine, serum phosphate, alkaline phosphatase, iPTH and cumulative parathyroid burden (Table 1).

Patients with primary hyperparathyroidism were more commonly female and exhibited higher total, as well as ionized, serum calcium concentrations, but lower iPTH levels compared with those diagnosed with SHPT. As expected, in the context of end-stage kidney disease and multiglandular involvement, serum creatinine, serum phosphate and cumulative parathyroid burden were increased in the SHPT group. Alkaline phosphatase levels were lower in patients with PHPT, most likely in relation to the comparatively lower degree of PTH excess. Although not of statistical significance, patients with SHPT were generally younger compared to those in the PHPT cohort.

In the following subsections, additional analyses were conducted separately within each group, according to histopathological lesion characteristic and imagistic findings.

### 3.2. Primary Hyperparathyroidism Subgroup

Patients diagnosed with primary hyperparathyroidism were stratified according to the histopathological diagnosis established after surgical excision of the specimen. The resulting cohort comprised 13 patients with parathyroid gland hyperplasia and 75 patients with parathyroid adenoma. Analysis of biochemical parameters revealed no statistically significant differences between groups in terms of total serum calcium (*p* = 0.6632), phosphate (*p* = 0.4854), creatinine (*p* = 0.3423) or alkaline phosphatase (*p* = 0.0869). Serum iPTH was the only biochemical variable that differed significantly, with higher values observed in patients with parathyroid adenoma (*p* = 0.0363) (Table A1). Cell type, growth patterns, adipose content, presence of calcifications, cystic degeneration, peripheral rim, and scintigraphic findings were subsequently analyzed according to lesion type (Table 2).

When parathyroid adenomas were compared with hyperplastic glands, the presence of a peripheral rim of normal parathyroid tissue was the only microscopic feature that reached statistical significance (*p* = 0.0151). On macroscopic examination, adenomas showed a significantly greater volume than hyperplastic lesions (1.25 vs. 0.45 mL *p* = 0.0079). Despite relatively subtle microscopic differences, adenomatous lesions were more frequently detected by parathyroid scintigraphy (*p* = 0.0082).

To further investigate the factors associated with negative scintigraphic findings, patients with PHPT were reclassified according to imaging results, and histopathological characteristics were reassessed (Table 3). A solid growth pattern was more frequently observed in lesions with a positive sestamibi uptake (*p* = 0.0118), whereas no other histopathological characteristics reached statistical significance.

**Figure 1:**
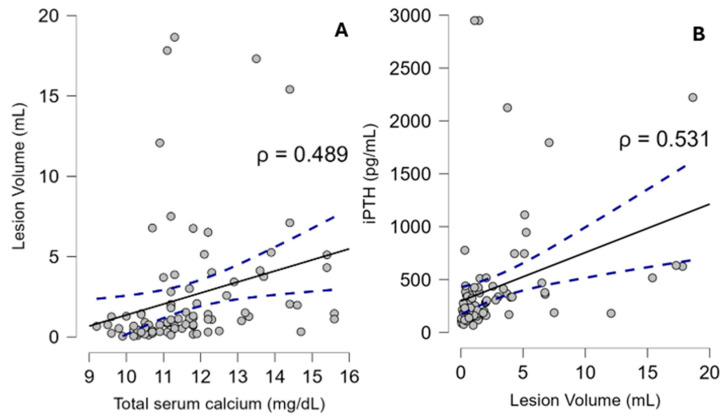
Spearman’s rank correlation for total serum calcium (**A**) and iPTH levels (**B**) and lesion volume in primary hyperparathyroidism.

A combined group analysis was performed using Spearman’s rank correlation to assess relationships involving lesion volume. Moderate but significant positive correlations were observed for lesion volume and total serum calcium (*rho* = 0.489, 95% CI: 0.312–0.634, *p* < 0.0001) as well as between lesion volume and iPTH (*rho* = 0.531, 95% CI: 0.362–0.666, *p* < 0.0001) (Figure 1). Logistic regression analysis including lesion type, growth pattern, lesion volume, total serum calcium and iPTH yielded a statistically significant model for predicting a positive parathyroid scintigraphy (*p* < 0.0004). Absence of a solid growth pattern (OR = 0.1501 *p* = 0.0046) and histopathological diagnosis of parathyroid hyperplasia (OR = 0.0841 *p* = 0.0017) emerged as the strongest predictors of negative scintigraphic detection. Although lesion volume remained statistically significant (*p* = 0.0102), total serum calcium and iPTH did not retain independent predictive value in the multivariable model (*p* = 0.3064 and 0.1719, respectively). The overall model demonstrated a moderate explanatory capacity (Nagelkerke R^2^ = 0.347), indicating that approximately 34.7% of the variability in outcome was explained by the included predictors. Discriminative performance was acceptable, with an AUC of 0.807. Overall sensitivity for positive sestamibi uptake was 77.27%, with higher detection rates for adenomatous lesions (82.66%) compared to hyperplastic glands (46.15%).

### 3.3. Secondary Hyperparathyroidism Subgroup

Given the multiglandular nature of secondary hyperparathyroidism, comparative analyses were performed using two derived variables: cumulative parathyroid burden, defined as the sum of the volumes of all excised lesions in each patient, and predominant histopathological pattern, established according to whether most glands were classified as nodular or diffuse hyperplasia. Biochemical assessments showed that patients with predominantly diffuse hyperplasia had significantly lower total and ionized serum calcium (*p* = 0.0002 and *p* = 0.0118, respectively). Higher alkaline phosphatase values were found to be more frequently associated with nodular hyperplasia (*p* = 0.0222) as was a greater cumulative parathyroid burden (1.16 vs. 2.63 mL *p* = 0.0007). No statistically significant differences were identified between these categories regarding age, gender, iPTH, serum phosphate or positive scintigraphic findings. Although fewer missed glands were recorded in patients with predominantly nodular hyperplasia, this difference narrowly failed to reach statistical significance (*p* = 0.0525) (Table 4).

**Figure 2:**
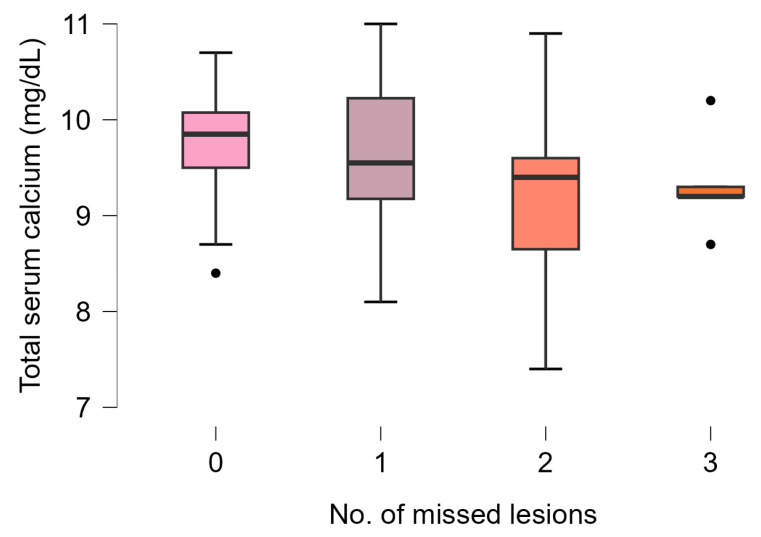
Boxplot for total serum calcium and number of omitted lesions in secondary hyperparathyroidism.

Across all SHPT cases, cumulative parathyroid burden showed a significant but weaker correlation with iPTH levels (rho = 0.393, *p* = 0.0005) compared to that observed in PHPT, suggesting that factors beyond lesion volume may influence the iPTH concentration in secondary disease. When assessed using the Kruskal–Wallis test, total serum calcium level tended to decrease as the number of scintigraphically missed lesions increased (Kruskal–Wallis H = 8.1641, df = 3, *p* = 0.0422) (Figure 2).

Individual lesion analysis is particularly relevant in SHPT, as different histopathological patterns may coexist whin an individual patient. A total of 219 lesions from 74 SHPT patients were evaluated and categorized according to the histological diagnosis. To minimize bias related to clustering of multiple glands withing individual patients, each excised lesion was treated as a separate analytical unit. At the lesion level, glands affected by nodular hyperplasia lesions were larger (*p* < 0.0001), presented more often with intralesional calcifications (*p* = 0.0209) and a reduced adipose content (*p* = 0.0019). Certain growth patterns, especially the pseudoadenomatous and nested arrangements, were rarely observed in diffuse hyperplasia compared to the nodular form (*p* = 0.0015 and *p* = 0.0031, respectively). When scintigraphic detection was assessed per lesion rather than per patient, diffuse hyperplasia was more often associated with a negative result (*p* < 0.0001) (Table 5).

**Figure 3:**
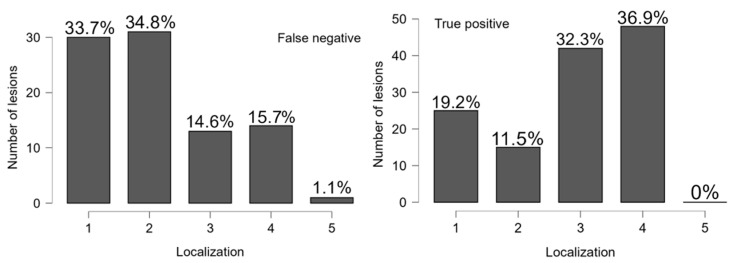
Frequency plot of anatomic lesion location according to scintigraphic results in secondary hyperparathyroidism. Left superior parathyroid gland—1; right superior parathyroid gland—2; left inferior parathyroid—3; right inferior parathyroid gland—4; ectopic—5.

When lesions were reclassified according to the scintigraphic result, larger lesions (*p* = 0.0001) and lesions with reduced stromal fat content (*p* = 0.0378) more frequently exhibited a positive sestamibi uptake (Table A2). Further comparison between detected and omitted lesions showed that superior anatomical position was associated with a lower likelihood of scintigraphic detection (*p* < 0.0001) (Figure 3) (Table A3). A significant association was also observed between gland localization and lesion type (${Χ}^{2}$ = 12.83, *p* = 0.0121). The proportion of lesions classified as diffuse hyperplasia varied across anatomical sites, with both left and right superior localization showing a higher prevalence of diffuse changes compared to both inferior localizations. Lesion volume also differed across anatomical quadrants (Kruskal–Wallis H = 13.96, df = 4, *p* = 0.007), with inferior glands exhibiting larger volume differences between left inferior gland and both superior glands, as well as between the right inferior gland and both superior glands (*p* < 0.05) (Table A4).

To determine factors influencing sestamibi detectability, logistic regression analysis was performed including iPTH values, lesion type, lesion volume, adipose content, anatomical position and pseudoadenomatous growth pattern. The resulting model was statistically significant (*p* < 0.0001) and showed acceptable discriminative performance with an AUC of 0.827. In the multivariate analysis, the left superior localization (OR = 0.2849, 95% CI: 0.1183–0.6862, *p* = 0.0051) and right superior localization (OR = 0.1017, 95% CI: 0.037–0.2770, *p* < 0.0001) were independently associated with a lower probability of sestamibi uptake. Lesion volume emerged as a significant positive predictor for uptake (OR = 3.2287, 95% CI: 1.8109–5.7566, *p* < 0.0001), whereas a diagnosis of diffuse hyperplasia showed a negative association that did not reach statistical significance in the combined model (OR = 0.3795, 95% CI: 0.1285–1.1212, *p* = 0.0796). Overall, the included variables accounted for approximately 40% of outcome variability (Nagelkerke R = 0.3966).

In the SHPT cohort, the complete patient-level localization rate of all pathological glands was 27.02%, varying from 9.09% in patients with predominantly diffuse hyperplasia and 43.18% in those with predominantly nodular hyperplasia. By contrast, when performance was assessed at the lesion level, ^99m^Tc-sestamibi scintigraphy demonstrated a sensitivity of 86.75%.

## 4. Discussion

In the present study, we evaluated factors associated with localizing ^99m^Tc-sestamibi dual-phase parathyroid scintigraphy, with particular emphasis on histopathological lesion characteristics and patient-level biochemical parameters. In our PHPT cohort, most lesions were parathyroid adenomas (85.2%), whereas parathyroid hyperplasia was less common. Among patients with parathyroid hyperplasia, two were diagnosed with multiple endocrine neoplasia type 1, one patient had a concomitant parathyroid adenoma and ten had isolated parathyroid hyperplasia without an associated genetic component. Most patients with PHPT were female, with a median age of 60 at diagnosis, which is consistent with the current literature regarding the age and sex distribution of PHPT [6,7,17]. Statistical comparison according to final histopathological diagnosis revealed no significant differences regarding biochemical parameters, except for higher PTH levels in patients with adenoma.

From a histological perspective, parathyroid adenomas more frequently exhibited a peripheral rim and showed greater lesion volume, while also being more commonly detected on parathyroid scintigraphy. Although lesion volume did not differ significantly between scintigraphically positive and negative lesions in the unadjusted comparison, it was independently associated with scintigraphic detectability when included in a multivariable logistic regression assessing factors contributing to sestamibi uptake; both lesion volume and a solid growth pattern were independently associated with scintigraphic detectability, whereas the histopathological diagnosis of hyperplasia was associated with reduced uptake of sestamibi. This apparent discrepancy may reflect a masking effect arising from the interrelationships among lesion volume and the included histopathological characteristics. In a previous study investigating parathyroid gland histopathological characteristics according to ^18^F-choline uptake, growth pattern was also identified as a relevant determinant, with solid architecture being associated with higher early-phase standard uptake values (SUVs) compared to other histological patterns. Late-phase SUVs were increased in lesions displaying a follicular growth pattern [18]. Because trabecular or nested architecture may imply less cellularity compared to a solid pattern, in line with aforementioned data, our study supports cellularity-related architectural features as contributors to positive scintigraphic findings. As a limitation, in our study, growth pattern was recorded qualitatively as present or absent rather than quantitatively assessed. Moreover, early- and late-phase uptake could not be expressed as numerical variables due to a different imaging technique which hinders further interpretation based on the identified growth pattern. Oxyphil cell content has been long proposed as an important factor influencing parathyroid lesion visualization. Given the mitochondrial density of oxyphil cells compared to chief cells, increased sestamibi uptake and retention has been hypothesized [21]. Several studies have reported higher rates of positive imaging in lesions with an abundant oxyphil content [22,23]. More recent data suggests that lesions with oxyphil content exceeding 30% are more likely to yield positive results [24]. Conversely, when using an oxyphil content over 25%, other authors found no significant differences between lesions with positive and negative imaging results [25]. In our cohort, pathology reports documented the presence or absence of specific cell types without quantifying percentage. Although this qualitative assessment did not reveal statistically meaningful differences between imaging groups, the lack of quantitative data for oxyphil cell content hinders further evaluation and represents one of the study limitations. This methodological limitation may have reduced the sensitivity of the analysis and could explain the discrepancy with previous studies reporting greater uptake in oxyphil-rich lesions.

Various morphometric features of parathyroid adenomas have been previously linked to be important predictors of sestamibi uptake. In a study including 252 patients, adenoma weight differed significantly between patients with positive and negative scans and demonstrated a good discriminatory performance, with an AUC = 0.81, a cut-off of 0.73 g yielding a sensitivity of 75% and a specificity of 82%. The same study also reported adenoma maximum diameter as a relevant parameter, although sensitivity decreased to 61% when a threshold of 18 mm was applied [21]. Adenoma weight had also been described as a positive predictor with an odds ratio of 4.477 for a positive dual-tracer SPECT/CT scan [26]. Similarly to weight, adenoma volume has been identified as one of the main factors influencing scintigraphy sensitivity in patients with primary, as well as secondary, hyperparathyroidism [18,25,27,28]. However, not all studies have confirmed this association; some authors found no relationship between adenoma volume and a positive scan, even after accounting for cystic degeneration [29]. In the present study, adenoma volume remained a significant predictor for a localizing study even when included in a multivariate logistic regression. In addition, lesion volume correlated positively with serum PTH, supporting the hypothesis that volumetric burden may serve as an indirect marker of hormonal hypersecretion.

Among patients with SHPT, stratification according to histopathological pattern revealed significant differences in total serum calcium, ionized calcium, alkaline phosphatase and parathyroid burden, whereas iPTH levels were comparable between subgroups. Nodular hyperplasia reflects a more advanced form of disease with significant alteration of parathyroid architecture and resistance to medical therapy. The lack of divergence in serum iPTH values might be explained, at least in part, by the greater variability of PTH compared to serum calcium, with the latter exhibiting a more consistent plasma value. Furthermore, the reduced responsiveness to medical therapy consistent with the nodular pattern implies concurrent treatment with increased doses of vitamin D analogous and calcimimetics leads to a more pronounced reduction in PTH compared to calcium concentration. In SHPT, individual patient classification and analysis is inherently complex because of multiglandular involvement and the potential coexistence of both lesion types within the same individual. In this study, patients were classified according to the predominant pattern identified across excised parathyroid glands. Accordingly, mixed diffuse and nodular changes in an individual might attenuate the differences observed between groups. Further analysis of patients in whom parathyroid scintigraphy failed to localize all excised glands identified total serum calcium as significantly different according to the number of missed glands. Specifically, calcium concentrations showed a decreasing trend with increasing number of omitted lesions. This observation indirectly supports previous reports showing that serum calcium levels are higher in patients with positive localization studies compared to patients with negative imaging [30].

When individual lesions were evaluated from a histopathological point of view, diffuse hyperplasia lesions were characterized by significantly smaller size, more abundant intralesional adipose tissue and lower cellularity, consisting in a nested growth pattern. By comparison, nodular hyperplasia more frequently exhibited intralesional calcifications. These findings are consistent with the pathophysiological alteration of parathyroid architecture induced by prolonged stimulation, while calcification and inhomogeneity have also been described in previous studies [11,31]. Beyond microscopic features, lesion volume and maximum diameter have previously been proposed as ultrasound predictors of progression from diffuse to nodular hyperplasia [14,32]. These findings may have practical implications for preoperative imaging in SHPT patients. Negative or equivocal planar scintigraphy, as well as identification of fewer pathological glands than would be expected from the overall clinical and biochemical severity of disease, should prompt consideration of additional imaging with US, SPECT/CT or, where available, ^18^F-FCH PET. Complementary imaging should also be considered when superior glands are not identified on a planar scintigraphy, with the aim of achieving more complete preoperative parathyroid mapping and reducing the risk of unnecessary reintervention or extensive surgical exploration.

Past studies have reported larger lesion volumes in patients with SHPT who had undergone localizing sestamibi imaging. Other lesion characteristics, including the presence of calcifications, cystic degeneration and intralesional adipose content, did not differ significantly according to scintigraphic detectability [28]. Our current findings confirm the association between larger lesion volumes and positive imaging, while also supporting the absence of a meaningful relationship between intralesional calcification or cystic changes and sestamibi uptake. Regarding adipose tissue, we report a significant difference between the two subgroups, with intralesional fat being more frequently identified in lesions lacking sestamibi uptake. However, although this parameter initially differed significantly between groups, when included in the multivariate model assessing predictors of a positive scan, the adipose did not retain its statistical significance. Variables that remained independently associated with scan positivity were lesion volume and anatomical position. In the generalized linear mixed-effects model, the patient-level random-intercept variance was effectively zero, indicating no detectable residual clustering of lesions originating from the same patient. In our SHPT cohort, superiorly located lesions were more likely to be missed, irrespective of laterality. This observation is supported by comparable findings in the literature [17,28]. On individual lesion analysis, planar ^99m^Tc-sestamibi scintigraphy showed a sensitivity of 86.75% for secondary hyperparathyroidism lesions, 77.2% sensitivity for lesions in patients with PHPT, and an overall sensitivity of 84.03% for the entire cohort. Although individual lesion sensitivity was satisfactory, the complete patient-level localization rate was only 27.02%. The complete localization rate was 9.09% among patients with predominantly diffuse hyperplasia and 43.18% among those with predominantly nodular disease. This marked difference highlights the difficulty in obtaining complete parathyroid mapping in the preoperative setting. While individual lesion sensitivity is commonly used to assess imaging performance, complete localization of all pathological glands is more relevant to surgical planning and biochemical cure in the setting of SHPT. Lesion-level sensitivity values are broadly consistent with previous evidence including a recent meta-analysis reporting a pooled sensitivity of 78.9% for planar ^99m^Tc-sestamibi scans [1].

## 5. Conclusions

The present study highlights the complexity of interpreting parathyroid scintigraphy in both primary and secondary hyperparathyroidism, emphasizing the influence of biochemical parameters, morphometric characteristics and histopathological features on lesion detectability. In PHPT, parathyroid adenomas represented the predominant pathological entity, biochemically expressing higher iPTH levels and greater volume compared to hyperplastic glands. Lesion volume, as well as the solid growth pattern, were identified as positive predictors for sestamibi uptake during scintigraphy. Moreover, the positive correlation between iPTH levels and lesion volume further supports the role of volumetric burden as an indirect marker of hormonal activity. In SHPT, imaging performance is strongly affected by the multiglandular nature of disease and by the coexistence of diffuse and nodular hyperplasia in individual patients. Nodular hyperplasia lesions were generally larger, more cellular and more frequently detected, whereas diffusely hyperplastic glands and upper quadrant lesions were linked to a lower likelihood of detection. These findings suggest that negative or incomplete scintigraphic studies should be interpreted cautiously, particularly in the preoperative assessment of patients with SHPT.

## Figures and Tables

**Table 1:** Subgroup characteristics and laboratory assessments.

Variable	PHPT (*n* = 88)	SHPT (*n* = 74)	*p*-Value
Age (years)	60 (51–68)	56.5 (48–66)	0.0887
Gender (female/male)	70/18	34/40	**<0.0001**
Serum creatinine (mg/dL)	0.8 (0.63–1.10)	9.2 (6.9–10.5)	**<0.0001**
Total serum calcium (mg/dL)	11.3 (10.7–12.25)	9.5 (9.1–10.0)	**<0.0001**
Ionized serum calcium (mg/dL)	4.95 (4.65–5.59)	4.22 (3.93–4.43)	**<0.0001**
Serum phosphate (mg/dL)	2.7 (2.3–3.0)	6.2 (5.4–7.5)	**<0.0001**
Alkaline phosphatase (U/L)	95 (74–136)	143 (103–264)	**0.0005**
iPTH (pg/mL)	230.9 (169.3–405.8)	2173 (1388–3018.3)	**<0.0001**
Parathyroid burden (mL)	1.25 (0.48–3.25)	2.41 (1.16–3.75)	**0.0213**

*n*, number of patients; iPTH, intact parathyroid hormone; data shown as median (IQR). *p*-values considered statistically significant (<0.05), as well as column titles were written in bold.

**Table 2:** Histopathological lesion characteristics and scintigraphic examination results according to lesion type in primary hyperparathyroidism.

Variable	Hyperplasia (*n* = 13)	Adenoma (*n* = 75)	*p*-Value
Lesion volume (mL)	0.45 (0.20–1.13)	1.25 (0.52–3.62)	**0.0079**
Peripheral normal parathyroid tissue rim	2/11 (15.3%)	40/35 (53.3%)	**0.0151**
Parathyroid scintigraphy (positive/negative)	6/7 (46.1%)	62/13 (82.6%)	**0.0082**
Chief cells (present/absent)	13/0 (100%)	67/7 (89.3%)	0.5878
Oxiphilic cells (present/absent)	3/10 (23%)	23/52 (30.6%)	0.7473
Solid growth pattern (present/absent)	9/4 (69.2%)	51/24 (68%)	1
Acinar growth pattern (present/absent)	8/5 (61.5%)	30/45 (40%)	0.2248
Trabecular growth pattern (present/absent)	2/11 (15.3%)	14/61 (18.6%)	1
Pseudoadenomatous growth pattern (present/absent)	0/13 (0%)	3/72 (4%)	1
Nested growth pattern (present/absent)	0/13 (0%)	6/69 (8%)	0.5859
Calcifications (present/absent)	0/13 (0%)	6/69 (8%)	0.5859
Cystic degeneration (present/absent)	3/10 (23%)	19/56 (25.3%)	1

*n*, number of patients; data shown as median (IQR). *p*-values considered statistically significant (<0.05), as well as column titles were written in bold.

**Table 3:** Histopathological lesion characteristics according to scintigraphic detectability in primary hyperparathyroidism.

Variable	Negative Lesions (*n* = 20)	Positive Lesions (*n* = 68)	*p*-Value
Solid growth pattern (present/absent)	9/11 (45%)	51/17 (75%)	**0.0118**
Lesion volume (mL)	1.21 (0.41–4.1)	1.08 (0.43–2.03)	0.5977
Chief cells (present/absent)	19/1 (95%)	61/6 (89.7%)	1.0000
Oxiphilic cells (present/absent)	5/15 (25%)	21/47 (30.8%)	0.7822
Acinar growth pattern (present/absent)	10/10 (50%)	28/40 (41.1%)	0.4862
Trabecular growth pattern (present/absent)	4/16 (20%)	12/56 (17.6%)	0.7529
Pseudoadenomatous growth pattern (present/absent)	20/0 (100%)	65/3 (95.5%)	1
Nested growth pattern (present/absent)	2/18 (10%)	4/64 (5.8%)	0.6152
Calcifications (present/absent)	2/18 (10%)	4/64 (5.8%)	0.6152
Cystic degeneration (present/absent)	7/13 (35%)	15/53 (22%)	0.2427

*n*, number of patients; data shown as median (IQR). *p*-values considered statistically significant (<0.05), as well as column titles were written in bold.

**Table 4:** Subgroup characteristics and laboratory assessments according to predominant histological pattern in secondary hyperparathyroidism.

Variable	Predominantly Diffuse Hyperplasia (*n* = 11)	Predominantly Nodular Hyperplasia (*n* = 63)	*p*-Value
Age (years)	55 (47.25–63.50)	58 (48.25–66.50)	0.533
Gender (female/male)	3/8	31/32	0.2082
Total serum calcium (mg/dL)	9.10 (8.05–9.27)	9.6 (9.20–10.07)	**0.0002**
Ionized serum calcium (mg/dL)	3.92 (3.50–4.16)	4.29 (4.02–4.45)	**0.0118**
Serum phosphate (mg/dL)	6.10 (5.70–7.50)	6.90 (4.80–7.62)	0.8587
Alkaline phosphatase (U/L)	98.5 (72.0–112.0)	154.0 (121.5–290.7)	**0.0222**
iPTH (pg/mL)	1619.0 (1375.7–2207.1)	2250.0 (1401.0–3076.1)	0.3123
Parathyroid burden (mL)	1.16 (0.36–1.48)	2.63 (1.52–4.05)	**0.0007**
Parathyroid scintigraphy (positive/negative)	1/10	44/19	0.2692
Number of missed lesions	2 (1–2)	1 (0–2)	0.0525

*n*, number of patients; data shown as median (IQR). *p*-values considered statistically significant (<0.05), as well as column titles were written in bold.

**Table 5:** Histopathological lesion characteristics and scintigraphic examination results according to histological pattern in secondary hyperparathyroidism.

Variable	Diffuse Hyperplasia (*n* = 29)	Nodular Hyperplasia (*n* = 190)	*p*-Value
Lesion volume (mL)	0.19 (0.12–0.34)	0.69 (0.37–1.34)	**<0.0001**
Chief cells (present/absent)	28/1 (96.5%)	188/2 (98.9%)	0.3023
Oxiphilic cells (present/absent)	19/10 (65.5%)	125/65 (65.7%)	0.9771
Solid growth pattern (present/absent)	19/10 (65.5%)	122/68 (64.2%)	0.8914
Acinar growth pattern (present/absent)	3/26 (10.3%)	26/164 (13.6%)	0.7746
Trabecular growth pattern (present/absent)	5/24 (17.2%)	20/170 (10.5%)	0.2906
Pseudoadenomatous growth pattern (present/absent)	3/26 (10.3%)	77/113 (40.5%)	**0.0015**
Nested growth pattern (present/absent)	4/25 (13.7%)	2/188 (1%)	**0.0031**
Calcifications (present/absent)	1/28 (3.4%)	40/150 (21%)	**0.0209**
Cystic degeneration (present/absent)	1/28 (3.4%)	18/172 (9.4%)	0.4803
Adipose content	2 (1–3)	1 (1–2)	**0.0019**
Scintigraphic examination (positive/negative)	7/22 (24.1%)	123/67 (64.7%)	**<0.0001**

*n*, number of patients; data shown as median (IQR). *p*-values considered statistically significant (<0.05), as well as column titles were written in bold.

## Data Availability

The original contributions presented in this study are included in the article. Further inquiries can be directed to the corresponding author.

## Abbreviations

The following abbreviations are used in this manuscript:

AUC	Area under the curve
CI	Confidence interval
CKD	Chronic kidney disease
df	Degrees of freedom
^18^F-FCH PET	Fluoro-choline positron emission tomography
GFR	Glomerular filtration rate
iPTH	Intact parathyroid hormone
PHPT	Primary hyperparathyroidism
PTH	Parathyroid hormone
SHPT	Secondary hyperparathyroidism
SUV	Standard uptake value

## Appendix A

**Table A1:** Demographics and laboratory assessments according to histological results in primary hyperparathyroidism.

Variable	Hyperplasia (*n* = 13)	Adenoma (*n* = 75)	*p*-Value
Age (years)	58 (43.25–69.25)	61 (52.25–67.75)	0.5024
Gender (F/M)	11/2	60/15	0.6988
Serum creatinine (mg/dL)	0.8 (0.60–0.97)	0.8 (0.65–1.08)	0.3423
Total serum calcium (mg/dL)	11.4 (10.6–11.8)	11.2 (10.7–12.3)	0.6632
Ionized serum calcium (mg/dL)	4.95 (4.65–5.27)	4.93 (4.65–5.63)	0.5999
Serum phosphate (mg/dL)	2.58 ± 0.49	2.71 ± 0.58	0.4854
Alkaline phosphatase (U/L)	75.5 (62–102)	96 (81–137)	0.0869
iPTH (pg/mL)	193.3 (136.7–211.4)	280.9 (169.3–424.8)	**0.0363**
Parathyroid scintigraphy (positive/negative)	6/7	62/13	**0.0082**

**Table A2:** Histopathological characteristics according to scintigraphic detectability in secondary hyperparathyroidism.

Variable	Negative Lesions (*n* = 89)	Positive Lesions (*n* = 130)	*p*-Value
Lesion type (diffuse/nodular)	22/67	7/123	**<0.0001**
Lesion volume (mL)	0.36 (0.19–0.66)	0.87 (0.45–1.58)	**<0.0001**
Chief cells (present/absent)	86/3	130/0	0.0657
Oxiphilic cells (present/absent)	61/28	83/47	0.5622
Solid growth pattern (present/absent)	54/35	87/43	0.0638
Acinar growth pattern (present/absent)	10/79	19/111	0.4697
Trabecular growth pattern (present/absent)	13/76	12/118	0.2202
Pseudoadenomatous growth pattern (present/absent)	33/56	47/83	0.8892
Nested growth pattern (present/absent)	3/86	3/127	0.6889
Calcifications (present/absent)	20/69	21/109	0.2402
Cystic degeneration (present/absent)	7/82	12/118	0.7250
Adipose content	1 (1–2)	1 (1–1)	**0.0378**

**Table A3:** Anatomical location of parathyroid lesions according to scintigraphic detectability in secondary hyperparathyroidism.

Anatomic location	Negative Lesions (*n* = 89)	Positive Lesions (*n* = 130)	*p*-Value
Left superior parathyroid (%)	33.70%	19.23%	
Right superior parathyroid	34.83%	11.53%	
Left inferior parathyroid	14.60%	32.30%	
Right inferior parathyroid	15.73%	36.90%	
Ectopic	1.12%	0%	
Overall comparison			**<0.0001**

**Table A4:** Subgroup analysis for total serum calcium according to the number of omitted lesions.

	Number of Patients	Total Serum Calcium—Median (IQR)	*p*-Value
No lesions missed	26	9.85 (8.40–10.10)	
1 missed lesion	20	9.55 (8.10–10.25)	
2 missed lesions	23	9.40 (8.62–9.60)	
3 missed lesions	5	9.20 (9.07–9.52)	
**Kruskal–Wallis H**			**0.0422**
